# Balancing the medical and social needs of children during the COVID‐19 pandemic

**DOI:** 10.5694/mja2.51804

**Published:** 2022-12-05

**Authors:** Keith Grimwood, Anne B Chang

**Affiliations:** ^1^ Menzies Health Institute Queensland Griffith University Gold Coast QLD; ^2^ Gold Coast Health Gold Coast QLD; ^3^ Australian Centre for Health Services Innovation Queensland University of Technology Brisbane QLD; ^4^ Queensland Children's Hospital Brisbane QLD; ^5^ Charles Darwin University Darwin NT

**Keywords:** COVID‐19

## Open access

Open access publishing facilitated by Griffith University, as part of the Wiley ‐ Griffith University agreement via the Council of Australian University Librarians.

## Competing interests

No relevant disclosures.

in reply: We thank Hyde^1^ for the interest in our Editorial, and for highlighting long COVID in children and adolescents.^2^ However, we contest the claim that our statement concerning symptoms in children and adolescents recovering from coronavirus disease 2019 (COVID‐19) inaccurately reflects the long COVID review.^3^ In that review, only two studies complied with the consensus research definition for long COVID, aligned with the World Health Organization, of symptoms persisting for more than 12 weeks.^4^ The difference in persistent symptoms between COVID‐19 cases and controls in one of these studies was 1.4% (95% confidence interval [CI], ‐2.2 to 5.0), and in the other it was 13.2% (95% CI, 10.9–15.5), but the low (13.4%) response rate risked sample bias. Indeed, even after including five additional case–control studies with symptoms persisting for more than 4 weeks, the median difference in symptoms was only 3.0%, leading the authors to state that “nearly all symptoms reported by children and adolescents infected with severe acute respiratory syndrome coronavirus 2 (SARS‐CoV‐2) are also reported in similar frequencies in those without evidence of infection”.^3^

Systematic reviews and meta‐analyses have also concluded that frequencies of most reported persistent symptoms were similar between SARS‐CoV‐2 cases and uninfected controls, and difficult to differentiate from symptoms following pandemic‐enforced school closures and lockdowns.^5^, ^6^ Overall, the studies were of poor quality, retrospective and subject to bias, and many lacked controls.^3^, ^5^, ^6^ Absence of an agreed case definition precluded prevalence estimates of long COVID in children and adolescents. Nevertheless, a meta‐analysis of five case–control studies found that persistent anosmia, headaches, cognitive impairment, and throat and eye discomfort were 2–8% more common following SARS‐CoV‐2 infection than in those without infection, but not fatigue, abdominal pain or myalgia.^5^

Prospective, longitudinal studies involving various age groups are needed to better characterise long COVID in children and adolescents. This includes recruiting suitable controls, employing a uniform case definition and individualised objective assessments. Finding controls who have never been infected will be challenging. Alternatives could be vaccinated individuals without prior proven infections or those with another proven viral infection. Detailed multidisciplinary pheno‐endotyping studies could then be performed to identify precision medicine‐based therapies.

Meanwhile, SARS‐CoV‐2 will continue to circulate. Vaccination and measures limiting airborne transmission, such as improving school indoor ventilation, will help reduce COVID‐19 and long COVID in children and adolescents.

## References

[1] Grimwood K , Chang AB . Balancing the medical and social needs of children during the COVID‐19 pandemic. Med J Aust 2022; 217: 299‐300. https://www.mja.com.au/journal/2022/217/6/balancing‐medical‐and‐social‐needs‐children‐during‐covid‐19‐pandemic 35970512 10.5694/mja2.51685 PMC9538464

[2] Hyde Z . Balancing the medical and social needs of children during the COVID‐19 pandemic [letter]. Med J Aust 2023; 218: 000‐000. 10.5694/mja2.51808 PMC9878100 36471917

[3] Zimmermann P , Pittet LF , Curtis N . The challenge of studying long COVID: an updated review. Pediatr Infect Dis J 2022; 41: 424‐426. 35213866 10.1097/INF.0000000000003502 PMC8997013

[4] Stephenson T , Allin B , Nugawela MD , et al. Long COVID (post‐COVID‐19 condition) in children: a modified Delphi process. Arch Dis Child 2022; 107: 674‐680. 35365499 10.1136/archdischild-2021-323624 PMC8983414

[5] Behnood SA , Shafran R , Bennet SD , et al. Persistent symptoms following SARS‐CoV‐2 infection amongst children and young people: a meta‐analysis of controlled and uncontrolled studies. J Infection 2022; 84: 158‐170. 10.1016/j.jinf.2021.11.011 PMC8604800 34813820

[6] Pellegrino R , Chiappini E , Licari A , et al. Prevalence and clinical presentation of long COVID in children: a systematic review. Eur J Pediatr 2022; 181: 3995‐4009. 36107254 10.1007/s00431-022-04600-x PMC9476461

